# Supplementary material for: Chiral Pseudogap Metal Emerging from a Disordered Van der Waals Mott Insulator 1T‐TaS2 − x Se x
Source: Adv Mater. 2025 Mar 23;37(18):2500287. doi: 10.1002/adma.202500287 (PMC12051792; doi:10.1002/adma.202500287)
Supplement: Supplementary file 1 — Supporting Information [file ADMA-37-2500287-s001.pdf]

ADVANCED MATERIALS

Supporting Information

for *Adv. Mater.*, DOI 10.1002/adma.202500287

Chiral Pseudogap Metal Emerging from a Disordered Van der Waals Mott Insulator 1*T*-$TaS_{2-x}Se_x$

*Hyunjin Jung, Jiwon Jung, ChoongJae Won, Hae-Ryong Park, Sang-Wook Cheong, Jaeyoung Kim, Gil Young Cho and Han Woong Yeom**

Supplementary Information for "Chiral Pseudogap Metal Emerging from a Disordered van der Waals Mott Insulator $1T$-$TaS_{2-x}Se_x$"

Hyunjin Jung,[1,2] Jiwon Jung,[1,2] ChoongJae Won,[1,3,4] Hae-Ryong Park,[1,2] Sang-Wook Cheong,[3,4,5] Jaeyoung Kim,[1] Gil Young Cho,[1,2,6] and Han Woong Yeom[1,2,*]

[1] *Center for Artificial Low Dimensional Electronic Systems, Institute for Basic Science (IBS), Pohang 37673, Republic of Korea*

[2] *Department of Physics, Pohang University of Science and Technology, Pohang 37673, Republic of Korea*

[3] *Laboratory for Pohang Emergent Materials, POSTECH, Pohang 37673, Republic of Korea*

[4] *MPPC-CPM, Max Planck POSTECH/Korea Research Initiative, Pohang 37673, Republic of Korea*

[5] *Rutgers Center for emergent Materials and Department of Physics and Astronomy, Rutgers University, NJ, USA*

[6] *Department of Physics, Korea Advanced Institute of Science and Technology, Daejeon 34141, Republic of Korea*

(Dated: February 26, 2025)

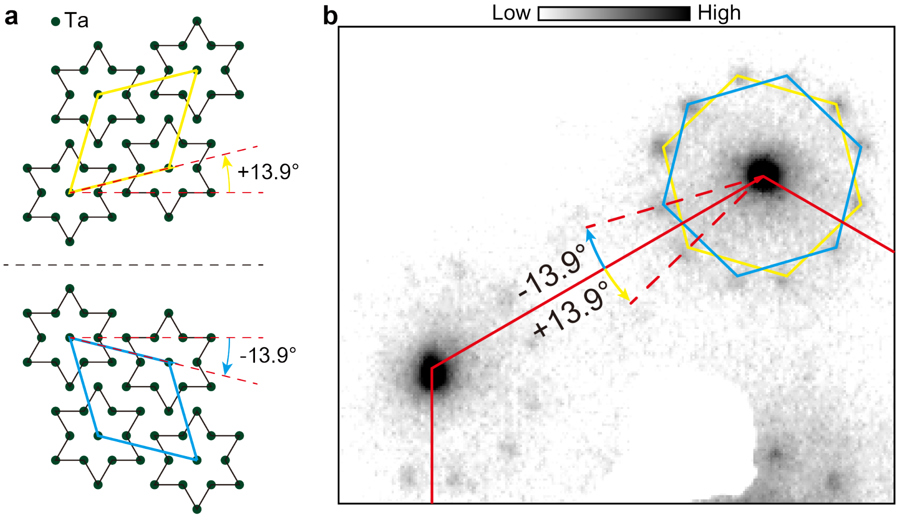

Fig. S1. (a), Schematics of the two ferro-rotational domains in 1T-TaSSe. (b), Grayscale LEED image for 1T-TaSSe (reproduced from Fig. 1e). The yellow and blue guidelines show the diffraction spots generated by two different CWD domains rotated by 27.8 degrees, matched well with FFT image of STM [1].

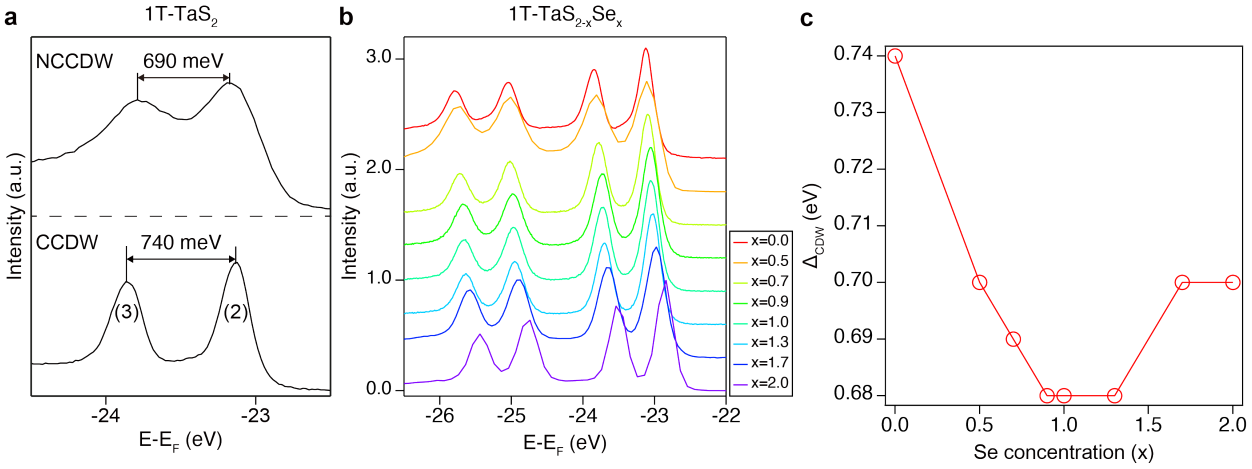

Fig. S2. (a), Ta $4f$ core level spectra of $1T$-TaS_2, showing energy splitting of 690 and 740 meV, respectively. Labeled (2) and (3) represent the contribution from the inner and outer Ta atoms within the David star cluster, respectively. (b), Ta $4f$ core level spectra of $1T$-$TaS_{2-x}Se_x$. The data for x=0.5 and x=2 were obtained using a photon energy of 200 eV, while the core level data for x=0.0, 0.7, 0.9, 1.0, 1.3, 1.7, and 2.0 were obtained using a photon energy of 65 eV. (c), The plot of Δ_{CDW} represents the average CDW splitting of the Ta $4f_{5/2}$ and Ta $4f_{7/2}$ core level as a function of Se concentration.

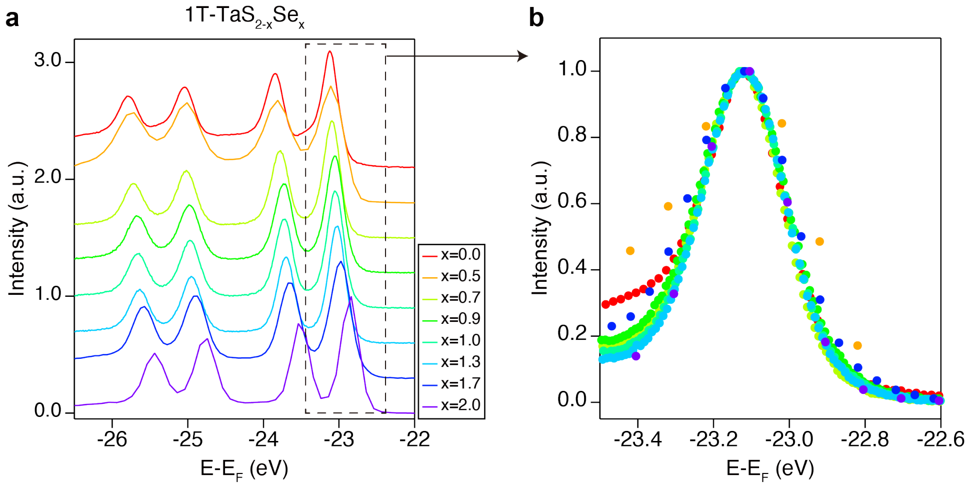

Fig. S3. (a) Ta 4f core-level spectra of $1T$-$TaS_{2-x}Se_x$ (reproduced from Fig. S2(b). (b) The same spectra as in (a), horizontally translated so that they overlap at the dashed line. The widths of the Ta 4f core level photoemission spectra are rather consistent for different sample compositions, while that of x=0.5 has a noticeably larger width. We thus interpret that such a width variation is not systematic but due to the different degrees of disorder onto the CDW order itself in the samples. In turn, these data suggest that the CDW order of the samples was maintained rather uniform over different compositions except for that with x=0.5. We used a different photon energy for x=0.5 and x=2.0 (200 eV) from other compositions (65 eV). Thus, the spectra at these two compositions can be broader than the others. However, since the width of the spectra for x=2.0 is consistent with those of others, we conclude the photon energy difference has a little effect on the total spectral broadening.

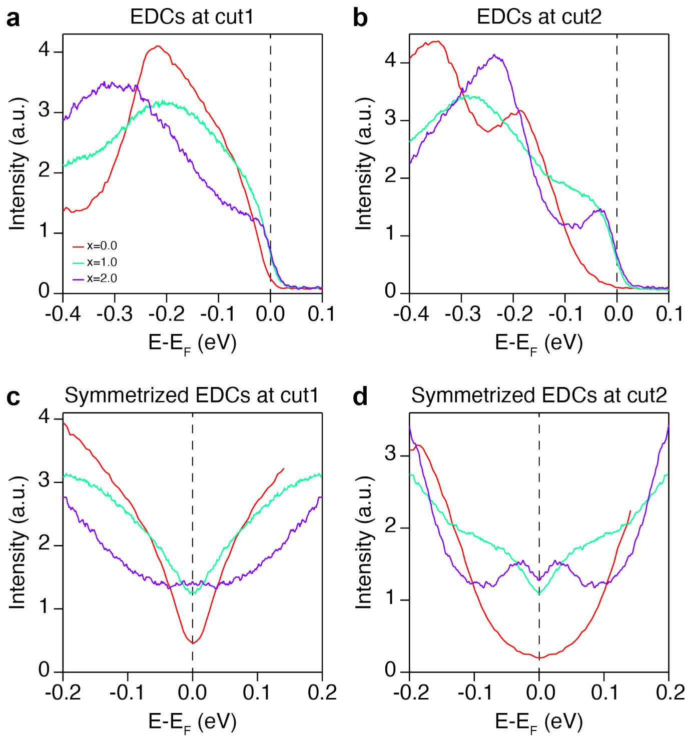

Fig. S4. Direct comparison of energy distribution curves (EDCs) and symmetrized EDCs for x=0, 1.0, and 2.0. (a) and (b) show the normalized EDCs for cut1 and cut2. (c) and (d) present the corresponding symmetrized EDCs.

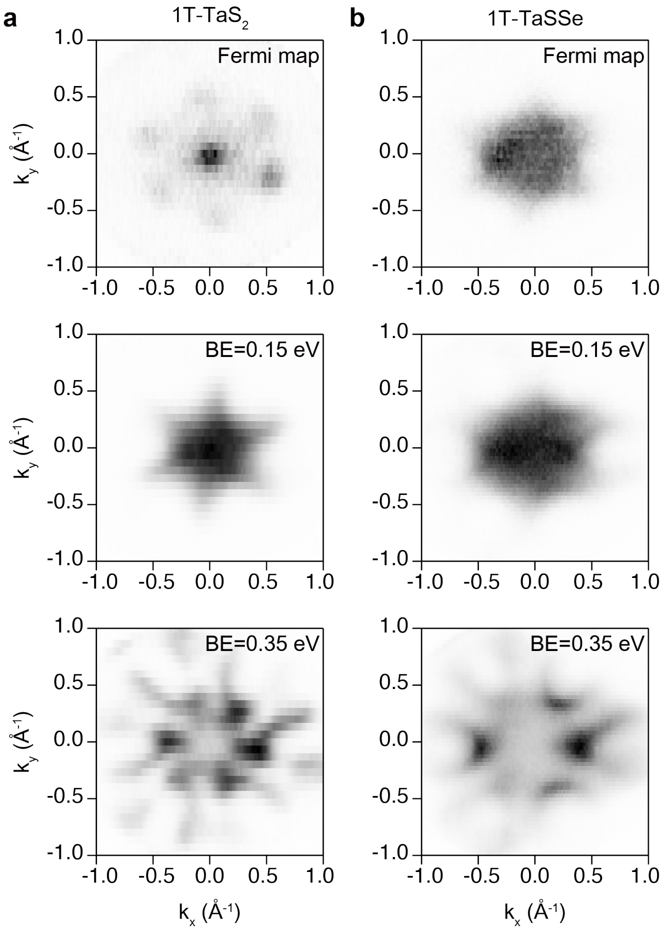

Fig. S5. Constant energy contour maps of (a), 1T-TaS_2 and (b), 1T-TaSSe at the binding energies of 0, 0.15 eV, and 0.35 eV.

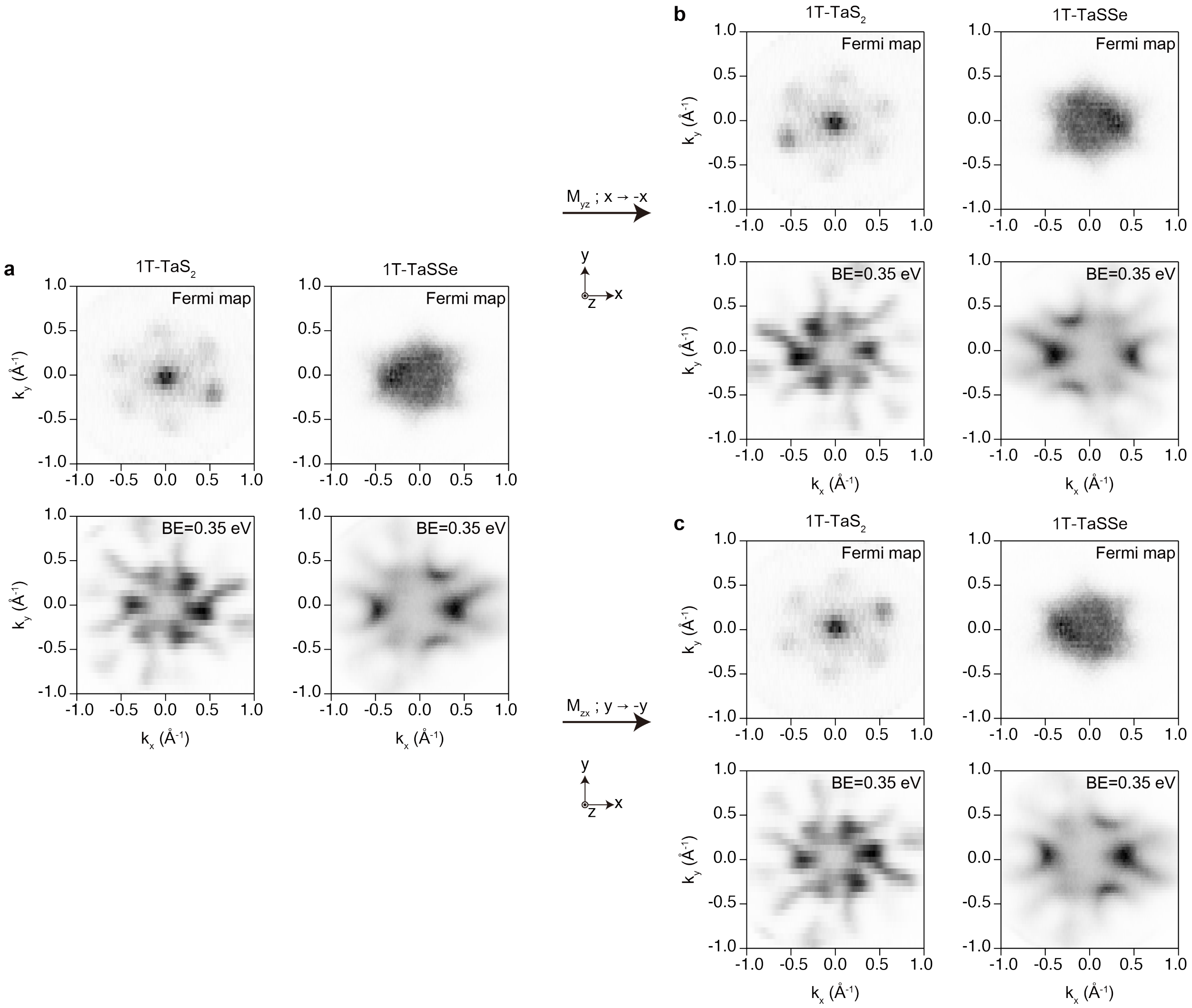

Fig. S6. Constant energy contours of $1T$-TaS_2 (left) and $1T$-TaSSe (right) at Fermi level and BE=0.35eV, respectively. The arrow labeled indicates the mirror-reflection operation along the k_x and k_y axis. The clear deviation from mirror symmetry in both materials highlights their chiral Fermi map topology.

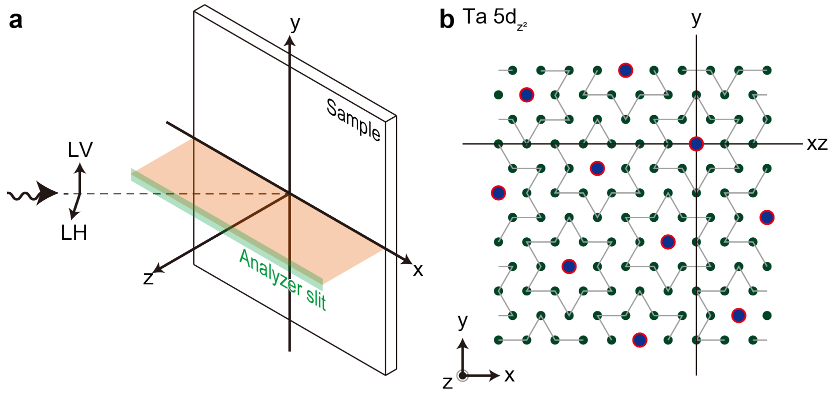

c

Beam polarization	Matrix element at Γ
x-pol	$\langle + \vert + \vert + \rangle \neq 0$
y-pol	$\langle + \vert - \vert + \rangle = 0$
z-pol	$\langle + \vert + \vert + \rangle \neq 0$

Fig. S7. Matrix element analysis for Ta $5d_{z^2}$ orbitals. (a), Schematics of ARPES setup for the polarization-dependent measurements. (b), illustration of the atomic arrangement for the Ta $5d_{z^2}$ orbital. (c), Table of the photoemission selection rule for xz parity at $\overline{\Gamma}$.

The one-electron dipole matrix element, known as dipole selection rule, can be described as follow [2, 3]:

$$M_{fi} \equiv \langle \phi_f | \boldsymbol{A} \cdot \boldsymbol{p} | \phi_i \rangle \,, \tag{1}$$

$$= \langle \phi_f | A_x p_x | \phi_i \rangle + \langle \phi_f | A_y p_y | \phi_i \rangle + \langle \phi_f | A_z p_z | \phi_i \rangle \,, \tag{2}$$

where ϕ_f and ϕ_f are the final and initial states of electron, $\boldsymbol{A}$ is electromagnetic vector potential of photon, and $\boldsymbol{p}$ is the electronic momentum operator. In our ARPES setup, the mirror plane is aligned along the xz-plane, resulting in both x- and z-polarization exhibiting even parity, while the y-polarization possesses odd parity. Give that the Ta $5d_{z^2}$ orbital is oriented along the z-axis, it maintains even parity with respect to the mirror plane. This symmetry consideration leasds to the matrix element table presented in Fig. S3(c). Specifically, only the y-polarized component carries odd parity, implying that electronic bands contributed from Ta $5d_{z^2}$ orbital are detectable exclusively under linear horizontal (LH) polarization. Consequently, the observation of these Ta $5d_{z^2}$-dominated bands is restricted to the LH polarization.

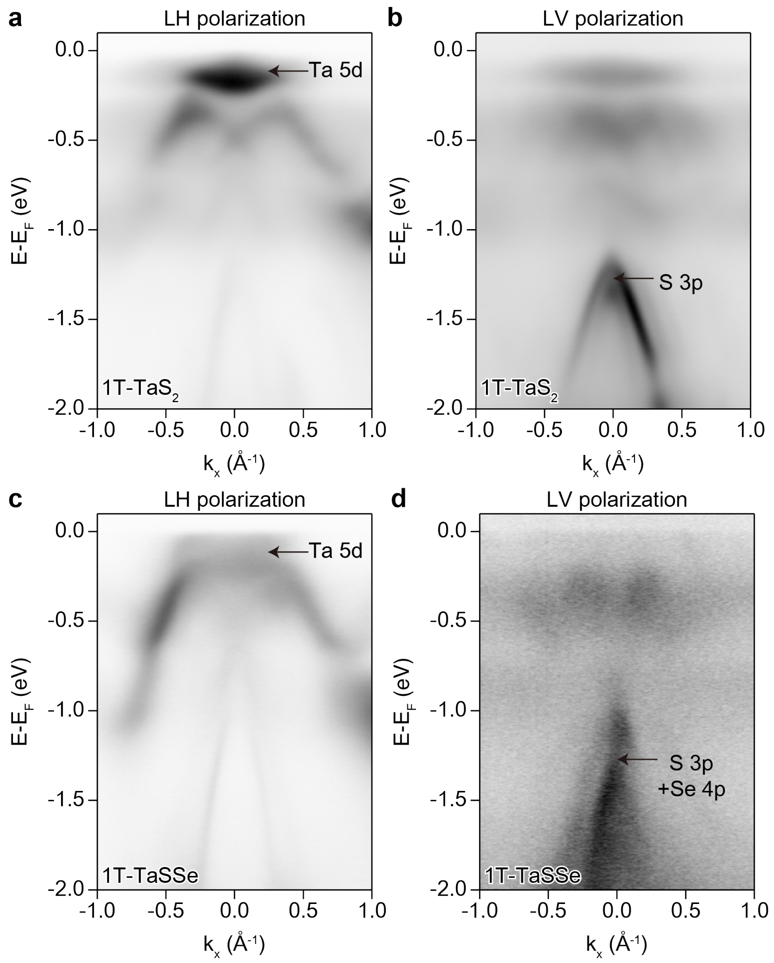

Fig. S8. Polarization-dependent ARPES spectra of $1T$-TaS_2 and $1T$-TaSSe. (a)-(b), ARPES intensity map with linear horizontal (LH) polarization and linear vertical (LV) polarization of $1T$-TaS_2 at 80 K, respectively. (c)-(d), ARPES intensity map with linear horizontal (LH) polarization and linear vertical (LV) polarization of $1T$-TaSSe at 70 K, respectively. The LH polarization primarily reveals the Ta 5d states, while the LV polarization highlights the contributions from the S 3p+Se 4p states.

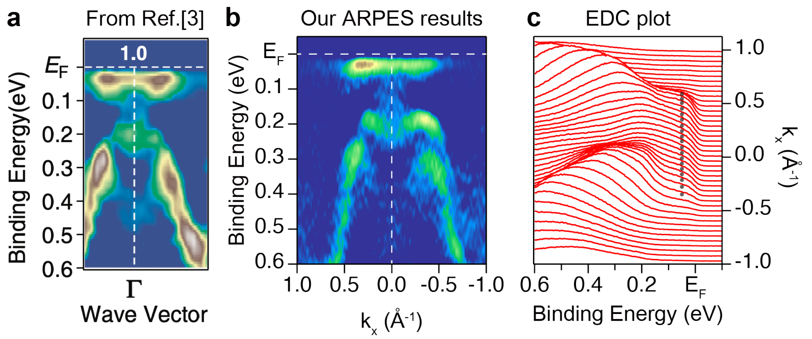

Fig. S9. Direct comparison of the electronic states of 1T-TaSSe between (a) Ref.[4] and (b) our second-derivative ARPES results. (c) shows the EDC plot of the raw data from (b). The gray dots indicate the pseudogap edges near the Fermi level (E_F).

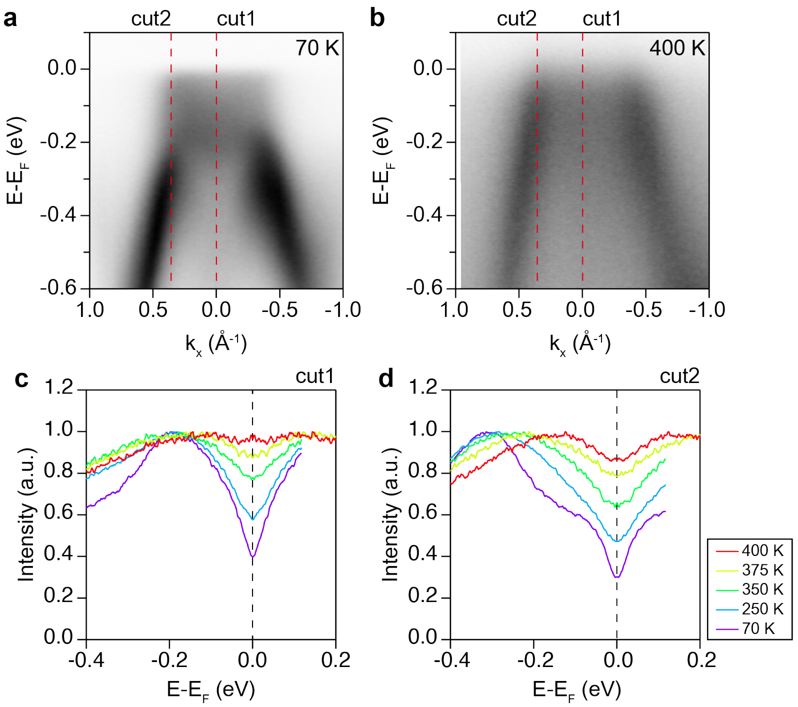

Fig. S10. Temperature-dependent ARPES measurements of 1T-TaSSe. (a-b), Electronic band structure at 70 K and 400 K, respectively. (c-d), Energy distribtuion curves (EDCs) at cut1 and cut2 with increasing temperature, respectively.

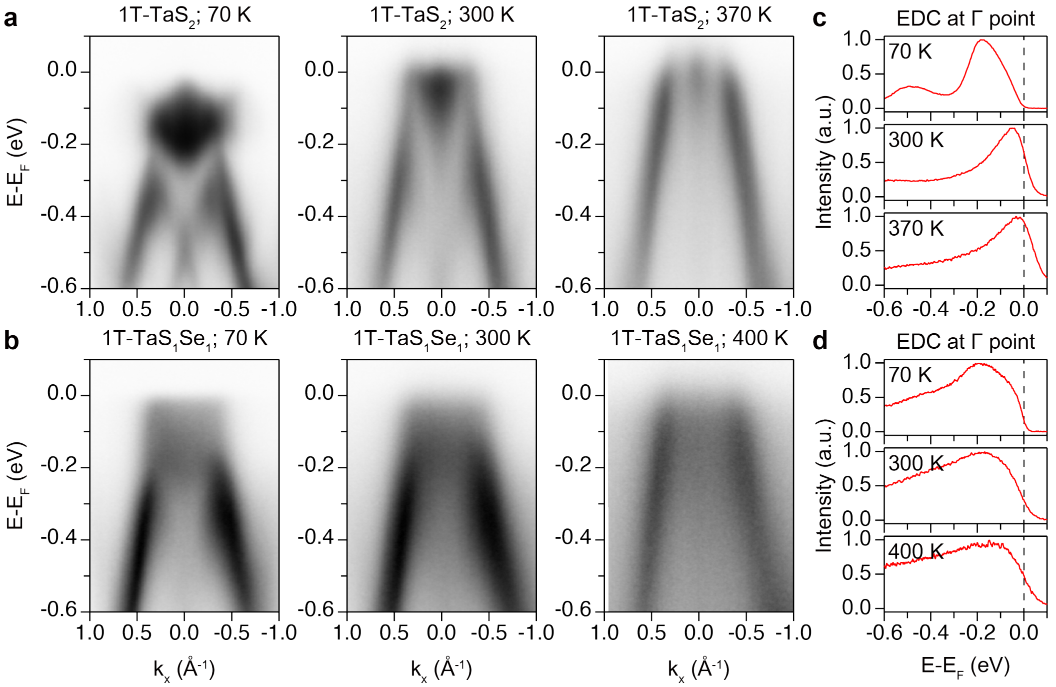

Fig. S11. Direct comparison of the temperature-dependent ARPES results between pristine 1T-TaS_2 and 1T-TaS_1Se_1. (a), Electronic band structure of pristine 1T-TaS_2 at 70 K, 300 K, and 370 K. (b), Electronic band structure of pristine 1T-TaS_1Se_1 at 70 K, 300 K, and 400 K. (c-d), Energy distribution curves (EDCs) at the Γ point.

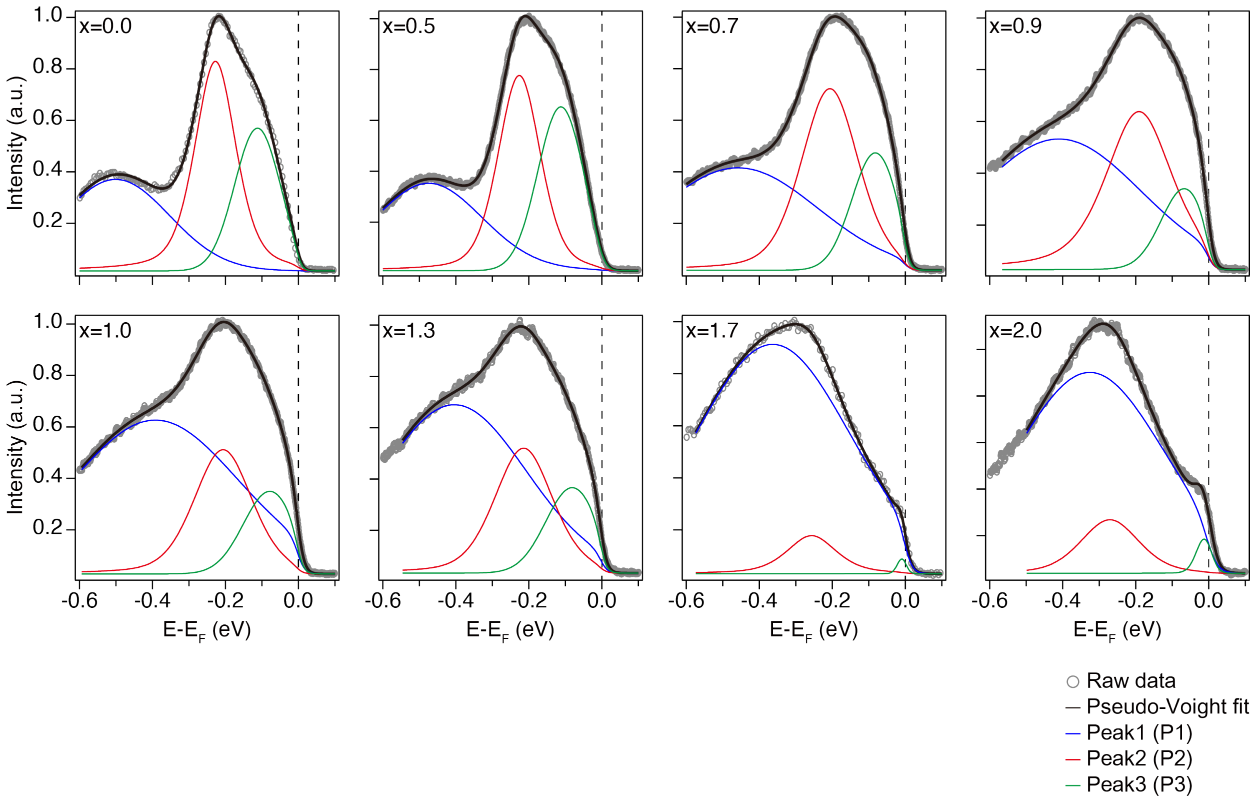

Fig. S12. Pseudo-Voigt fits of EDC spectra at $\overline{\Gamma}$ point for x=0.0, 0.5, 0.7, 0.9, 1.0, 1.3, 1.7 and 2.0.

* yeom@postech.ac.kr

[1] J. Gao, J. W. Park, K. Kim, S. K. Song, H. R. Park, J. Lee, J. Park, F. Chen, X. Luo, Y. Sun, *et al.*, Pseudogap and weak multifractality in 2d disordered mott charge-density-wave insulator, Nano Letters **20**, 6299 (2020).

[2] A. Damascelli, Z. Hussain, and Z.-X. Shen, Angle-resolved photoemission studies of the cuprate superconductors, Reviews of modern physics **75**, 473 (2003).

[3] K. Fukutani, R. Stania, C. Il Kwon, J. S. Kim, K. J. Kong, J. Kim, and H. W. Yeom, Detecting photoelectrons from spontaneously formed excitons, Nature Physics **17**, 1024 (2021).

[4] R. Ang, Y. Miyata, E. Ieki, K. Nakayama, T. Sato, Y. Liu, W. Lu, Y. Sun, and T. Takahashi, Superconductivity and bandwidth-controlled mott metal-insulator transition in 1 t-tas 2- x se x, Physical Review B **88**, 115145 (2013).
